# Facile One-Pot Preparation of Polypyrrole-Incorporated Conductive Hydrogels for Human Motion Sensing

**DOI:** 10.3390/s24175814

**Published:** 2024-09-07

**Authors:** Zunhui Zhao, Jiahao Liu, Jun Lv, Bo Liu, Na Li, Hangyu Zhang

**Affiliations:** 1 Liaoning Key Lab of Integrated Circuit and Biomedical Electronic System, School of Biomedical Engineering, Faculty of Medicine, Dalian University of Technology, Dalian 116024, China; zhao_zh@mail.dlut.edu.cn (Z.Z.); ljh2356486741@163.com (J.L.); lbo@dlut.edu.cn (B.L.); lina316@dlut.edu.cn (N.L.); 2 School of Mechanics and Aerospace Engineering, Dalian University of Technology, Dalian 116024, China; lvjun@dlut.edu.cn

**Keywords:** conductive hydrogel, one-pot preparation, polypyrrole, hydrogen peroxide, strain sensor, human motion sensing

## Abstract

Conductive hydrogels have been widely used in soft robotics, as well as skin-attached and implantable bioelectronic devices. Among the candidates of conductive fillers, conductive polymers have become popular due to their intrinsic conductivity, high biocompatibility, and mechanical flexibility. However, it is still a challenge to construct conductive polymer-incorporated hydrogels with a good performance using a facile method. Herein, we present a simple method for the one-pot preparation of conductive polymer-incorporated hydrogels involving rapid photocuring of the hydrogel template followed by slow in situ polymerization of pyrrole. Due to the use of a milder oxidant, hydrogen peroxide, for polypyrrole synthesis, the photocuring of the hydrogel template and the growing of polypyrrole proceeded in an orderly manner, making it possible to prepare conductive polymer-incorporated hydrogels in one pot. The preparation process is facile and extensible. Moreover, the obtained hydrogels exhibit a series of properties suitable for biomedical strain sensors, including good conductivity (2.49 mS/cm), high stretchability (>200%), and a low Young’s modulus (~30 kPa) that is compatible with human skin.

## 1. Introduction

In recent decades, the healthcare industry has experienced a period of rapid growth, benefiting from significant advances in information technology and electronics science. With the increasing demands to monitor various physiological signals, it is of vital importance to develop advanced bioelectronics and advanced preparation processes. As a potential candidate in the field of bioelectronics, conductive hydrogels exhibit a series of advantages, including biocompatibility, excellent stretchability, versatile designability and processability, and low cost of production [1,2,3]. Nevertheless, it is still a challenge to achieve a balance between stable sensing performance and a simple preparation process during the fabrication of hydrogel-based sensors.

Conductive polymers (CPs) like polypyrrole (PPy), polyaniline (PANI), and poly (3,4-ethylenedioxythiophene) (PEDOT) have been widely used for the preparation of conductive hydrogels owing to their intrinsic conductivity [4] and high biocompatibility [5,6,7,8,9,10]. Currently, a common strategy for preparing conductive polymer hydrogels (CPHs) involves separating the polymerizations of the hydrogel network and the conductive polymer. Basically, there are two technical routes that can be pursued to fulfill the aforementioned strategy. The first route entails immersing the hydrogel template into the monomer solution of CPs, thereby facilitating the growth of CPs along the hydrogel network [5,11,12,13,14,15,16,17,18,19]. The alternative route involves hydrogel gelation in a suspension of CP particles [20,21,22,23,24,25,26,27,28,29,30]. The former strategy allows for the construction of CPHs with high CP contents. However, the operational complexity of the immersion preparation method may limit the application of CPHs. A typical immersion preparation process comprises two steps, each of which is time-consuming and requires immersion in a particular solution. For example, Zhang et al. constructed a highly conductive and tough hydrogel with uniformly distributed PPy nanospheres [14]. The first step involved immersing the material in pyrrole for 24 h, while the second step required an additional 12 h of immersion in ferric chloride. Due to the insolubility of CPs in water, it is also a challenge to directly disperse CP particles evenly in the hydrogel precursor solution. Accordingly, modifications are frequently implemented with the objective of improving the dispersibility of CPs. To illustrate, Wang et al. developed a synthesis strategy for the preparation of water-soluble PPy [21], which was subsequently employed in the fabrication of hydrogel-based human–machine interfaces. In contrast to the aforementioned approach, Han et al. elected to pursue an alternative methodology for modifying CPs [22]. The initial step involved the extraction of cellulose nanofiber (CNF) from wood pulp [31]. This was followed by the in situ polymerization of polyaniline using CNF as a template. Hence, in order to prepare CPHs with good conductivity, high biocompatibility, and excellent mechanical properties, complicated treatments are usually unavoidable.

A one-pot preparation method is commonly used in hydrogel construction due to its convenience and moldability. Nevertheless, it was rarely employed to prepare CPHs. In addition to the poor solubility of conductive polymers, there are several conflicts between the gelation of hydrogels and the polymerization of CP monomers. In order to avoid the decrease in conductivity of CPs due to their aggregation during the thermal curing of the hydrogel template, a low-temperature or room-temperature environment is required [4,32]. Therefore, it is more eligible to prepare hydrogels through photocuring. Unfortunately, the darkening of the solution caused by the polymerization of CP monomers is an obstacle to photocuring, while the commonly used oxidants of CP monomers either promote (e.g., ammonium persulfate) or impede (e.g., iron (III)) hydrogel polymerization. Consequently, it is challenging to initiate the polymerization of CP monomers after the photocuring of hydrogel matrices without additional operations.

Herein, we report a facile and extensible one-pot processing procedure for the preparation of CPHs with good conductivity and stretchability. The use of a milder oxidant, hydrogen peroxide (H_2_O_2_), enables the photochemistry synthesis of CPHs in one pot. The procedure comprises three steps, involving the preparation of the precursor solution, the rapid photocuring of the hydrogel template, and the long-time polymerization of pyrrole. The conductivity and the mechanical performance of the hydrogels were sufficient for human motion sensing as strain sensors.

## 2. Materials and Methods

### 2.1. Materials

Sulfobetaine methacrylate (SBMA, 98%), N,N′-methylenebisacrylamide (MBAA, 99%), acrylamide (AAm, >99%), acrylic acid (AA, >99%), ammonium persulfate (APS, 98.5%), phytic acid (PA, 50%), pyrrole (Py, 99%), aniline (Ani, ≥99.9%), hydroxyethyl methacrylate (HEMA, 96%), and lithium phenyl (2,4,6-trimethylbenzoyl) phosphinate (LAP, ≥98%) were all purchased from Macklin (Shanghai, China). Hydrogen peroxide (H_2_O_2_, 30%) was ordered from Tianjin Kemiou Chemical Reagent Co., Ltd (Tianjin, China). Photo-initiator Irgacure 2959 (2959, 96%) was supplied by BASF (Ludwigshafen, Germany). Deionized water (DI) was prepared using a Milli-Q ultrapure water system supplied by Merck Millipore (Burlington, MA, USA).

### 2.2. Recipes of the Hydrogels

**Figure 1:**
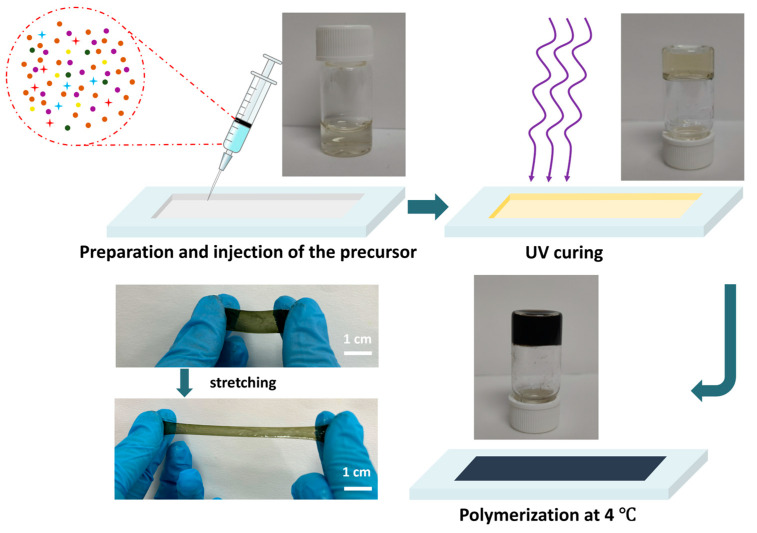
Schematic diagram for the preparation process of the CPHs through the one-pot method.

Figure 1 is a schematic diagram detailing the preparation of CPHs through our one-pot method. Briefly, hydrogel monomer SBMA, cross-linker MBAA, photo-initiator 2959, PA, H_2_O_2_, and pyrrole were dispersed in deionized water by ultrasonication to prepare the precursor solution. Subsequently, the precursor solution was injected into the mold, followed by ultraviolet curing for 15 min to obtain a transparent light-yellow hydrogel due to the existence of pyrrole and PA molecules. Finally, the cured hydrogel was placed under 4 °C for the mild growth of PPy chains, resulting in a black hydrogel colored by PPy.

For the recipes of hydrogels without incorporated PPy, namely PSBMA hydrogels and PA/PSBMA hydrogels, 2959 (0.8 wt%), MBAA (0.3 wt%), and various amounts of PA solution (corresponding to 0 wt%, 0.8 wt%, 1.6 wt%, 2.4 wt%, and 3.6 wt%) were added into 1 mL of SBMA solution (40 wt% in DI). The added powders were completely dissolved by ultrasonication for 10 min. It is noted that a low temperature and dark environment were essential for the prevention of gelation.

For the recipes of PPy/PA/PSBMA hydrogels, pyrrole and H_2_O_2_ with specific concentrations (molar ratio was set as 1:1) were additionally mixed with the above solution. After 15 min of ultraviolet curing in a mold, the cured hydrogel was placed in a fridge at 4 °C for 12 h to enable the polymerization of the PPy chains.

For another recipe of the hydrogels derived from our one-pot program, namely the PPy/PA/P (AAm-co-AA) hydrogel, it could be easily prepared by replacing only part of the materials without altering the fabrication program. The cross-linker MBAA, photo-initiator LAP, doping acid PA, pyrrole, and oxidant H_2_O_2_ with specific amounts (detailed contents are given in Table S1, in ESI) were mixed with a solution containing AAm (18 wt%) and AA (2 wt%).

### 2.3. Characterization of Hydrogels

Fourier transform infrared (FT-IR) spectra were acquired using a Thermo Scientific Nicolet IS50 (Waltham, MA, USA). X-ray photoelectron spectroscopy (XPS) spectra were acquired using a Thermo Scientific ESCALAB Xi+ (Waltham, MA, USA). All hydrogel samples used for FT-IR and XPS characterizations were lyophilized using a BIOCOOL vacuum freeze dryer FD-1A-BD (Beijing, China).

Ultraviolet–visible (UV–Vis) spectra were acquired using a spectrophotometer Jenway 7305 (Staffordshire, UK) in the wavelength range from 400 to 1000 nm, with a resolution of 1 nm and a quartz plate as the substrate. The precursor solution of the PPy/PA/PSBMA hydrogel was scanned every 1 h.

The electrical resistance values of the hydrogels were recorded on a LinkZill TruEbox-01RC (Hangzhou, China). In order to measure the conductivity, the hydrogels with a size of 40 mm × 10 mm × 3 mm were attached between two pieces of copper tape. The conductivity (*σ*) can be calculated through Equation (1):

(1) $$\sigma =\frac{d}{R\cdot S}$$

where *d* refers to the length, *R* refers to the electrical resistance, and *S* refers to the cross-sectional area.

All tensile tests on the hydrogels were conducted on a motorized tensile machine ZHIQU ZQ-990B (Dongguan, China). The hydrogels with a size of 40 mm × 10 mm × 3 mm were fixed using grippers, and the original length (*L*_0_) of the strain sensor was determined as 30 mm. The strain (*ε*) was calculated using Equation (2):

(2) $$\varepsilon =\frac{\Delta L}{L_{0}}=\frac{L-L_{0}}{L_{0}}$$

The gauge factor (*GF*) of a strain sensor was defined using Equation (3):

(3) $$GF=\frac{\Delta R/R_{0}}{\varepsilon }$$

where *R*_0_ refers to the initial electrical resistance (i.e., the first recorded resistance value during measuring), and Δ*R* refers to the difference between *R*_0_ and the current electrical resistance value.

The response and recovery times were defined as the times required to reach the final change after a rapid 10% strain loading and unloading [33].

The electrical resistance and strain curves were obtained by recording the ΔR/R_0_ for every 5% strain in the range of 0~200%. Moreover, three types of tests were conducted to examine the response stability of the hydrogel as a strain sensor:

1. The frequency was fixed at 0.5 Hz, while the strains were varied at 20%, 40%, and 60%.
2. The strain was fixed at 20%, while the frequencies were varied at 0.25 Hz and 0.5 Hz.
3. The strain and frequency were fixed at 15% and 0.5 Hz, respectively, with 100 cycles.

Furthermore, an equipped strain sensor for motion detection was fabricated by sandwiching the hydrogel between two pieces of 3M VHB tape (MN, USA) with copper tapes as conducting wires.

## 3. Results and Discussion

### 3.1. Synthesis of PPy/PA/PSBMA Hydrogels

**Figure 2:**
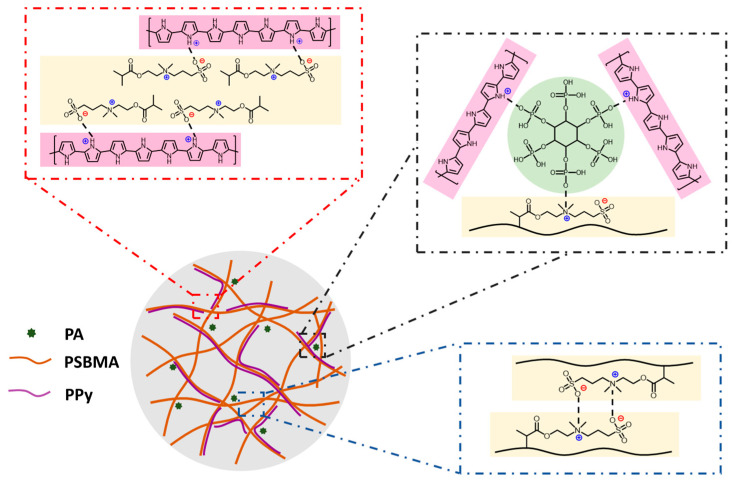
Various interactions among components of the PPy/PA/PSBMA hydrogel.

**Figure 3:**
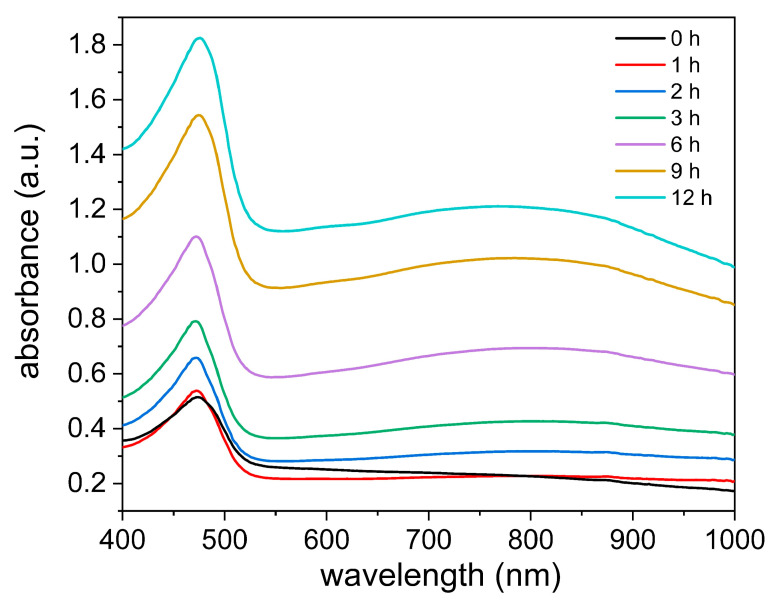
UV–Vis spectra of PPy/PA/PSBMA hydrogel during PPy polymerization.

Zwitterionic SBMA was first applied to construct the hydrogel matrix. As shown in Figure 2, abundant interactions exist among PA molecules, PPy chains, and the zwitterionic groups of PSBMA, enhancing the mechanical strength of the hydrogel. In addition to providing noncovalent cross-linking, as an organic doping acid with six dihydrogen phosphate, PA is more efficient in improving the conductivity of CPs compared with inorganic acids [34,35,36]. Moreover, H_2_O_2_ was selected as an oxidizing agent for the in situ polymerization of PPy chains without disturbing the hydrogel gelation. Compared to commonly used oxidizing agents, such as ammonium persulfate (APS), potassium persulfate, and ferric chloride (FeCl_3_), H_2_O_2_ would not promote or impede hydrogel polymerization and could oxidize pyrrole in a more gentle manner [37] to avoid PPy aggregation. The UV–Vis spectra of the as-prepared hydrogel during PPy polymerization are shown in Figure 3. The hydrogel exhibits a very low absorbance within the first hour, indicating a highly transparent state, which is a necessary condition for the photocuring of the hydrogel matrix.

**Figure 4:**
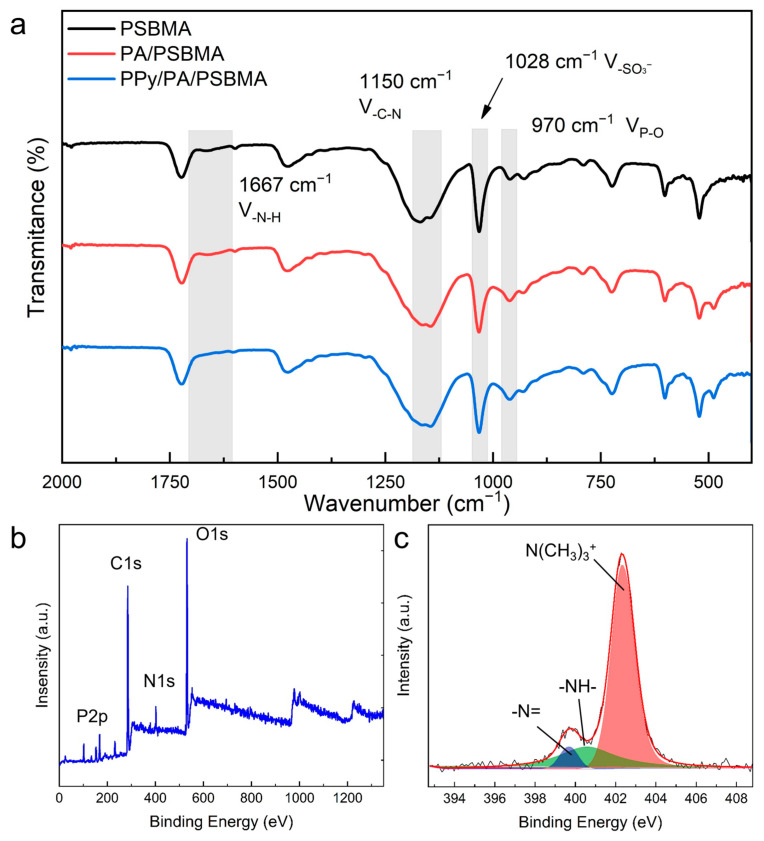
FT-IR and XPS spectra of the hydrogels. (**a**) FT-IR spectra of the PSBMA hydrogel, PA/PSBMA hydrogel, and PPy/PA/PSBMA hydrogel. (**b**) XPS spectra of the PPy/PA/PSBMA hydrogel. (**c**) Magnified spectra of the N1s area.

The FT-IR spectra of the PSBMA hydrogel, PA/PSBMA hydrogel, and PPy/PA/PSBMA hydrogel are shown in Figure 4a. The peaks around 970 cm^−1^, 1028 cm^−1^, and 1150 cm^−1^ correspond to the P-O bonds of the PA, -SO_3_^−^ groups on PSBMA, and C-N bonds, respectively. In addition, in the spectra of the PPy/PA/PSBMA hydrogel, the peak around 1667 cm^−1^ is lower than that in other spectra, indicating that the interactions among the PA, PPy, and PSBMA molecules influence the N-H bonds on PSBMA [18,38,39,40]. XPS was employed to further demonstrate the successful photochemistry synthesis of the PPy/PA/PSBMA hydrogel. The C1s, N1s, O1s, and P2p peaks are apparent in Figure 4b. Figure 4c displays the magnified spectra of the N1s region, and a strong peak appears at ca. 402.7 eV, assigned to the N (CH_3_)_3_^+^ moieties on PSBMA [40]. The peaks at ca. 401.1 eV and ca. 399.5 eV belong to the -NH- and -N= bonds, respectively, indicating the formation of PPy [41,42].

### 3.2. Electrical and Mechanical Properties of PPy/PA/PSBMA Hydrogels

The molar ratio of pyrrole to H_2_O_2_ was set at 1:1. As displayed in Table 1, when the concentration of PA was maintained at a constant level, the conductivity of the hydrogels incorporating PPy was approximately twice that of the hydrogels without PPy. The enhanced conductivity provides compelling evidence for the formation of intrinsically conductive networks of PPy. It is worth noting that the conductivity of PA/PSBMA hydrogels increased in proportion to the dosage of PA, owing to the increase in ions supplied by PA. Correspondingly, the conductivity of PPy/PA/PSBMA hydrogels exhibited a peak value of 2.49 mS/cm, followed by a declined conductivity with a constant increase in the initial pyrrole content. The reduction in conductivity at higher pyrrole concentrations is likely attributable to the aggregation of PPy, which may have influenced the formation of conductive networks.

**Figure 5:**
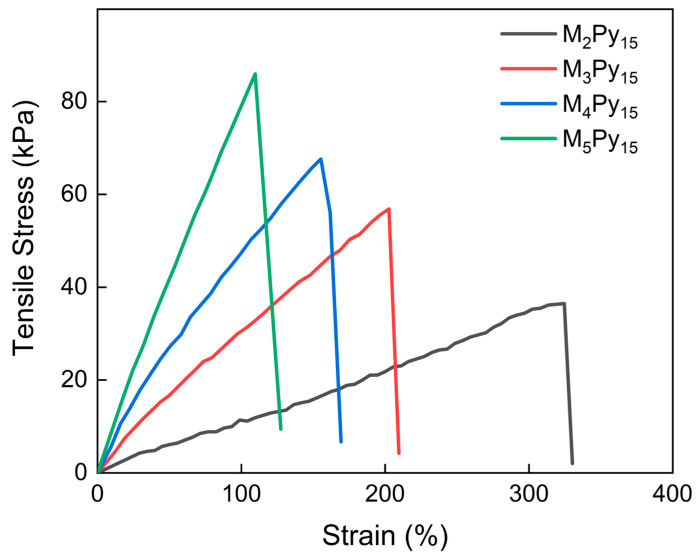
The tensile stress and strain curves of the PPy/PA/PSBMA hydrogels with different cross-linking degrees.

Furthermore, to obtain hydrogels with a tunable mechanical performance, the concentration of cross-linker MBAA was varied, and the tensile stress and strain curves are shown in Figure 5. The detailed material compositions and results are listed in Table 2. It is shown that the Young’s modulus of the hydrogels increased with increasing MBAA, and the hydrogels became harder and more brittle, exhibiting an increase in tensile stress at break as well as a decrease in elongation at break. The wide and adaptable range of the Young’s modulus provided by PPy/PA/PSBMA hydrogels is highly compatible with the specifications of skin-attached strain sensors (0.5–100 kPa [43]), rendering them suitable for the majority of human motion detection scenarios.

### 3.3. Sensing Performance of PPy/PA/PSBMA Strain Sensors

**Figure 6:**
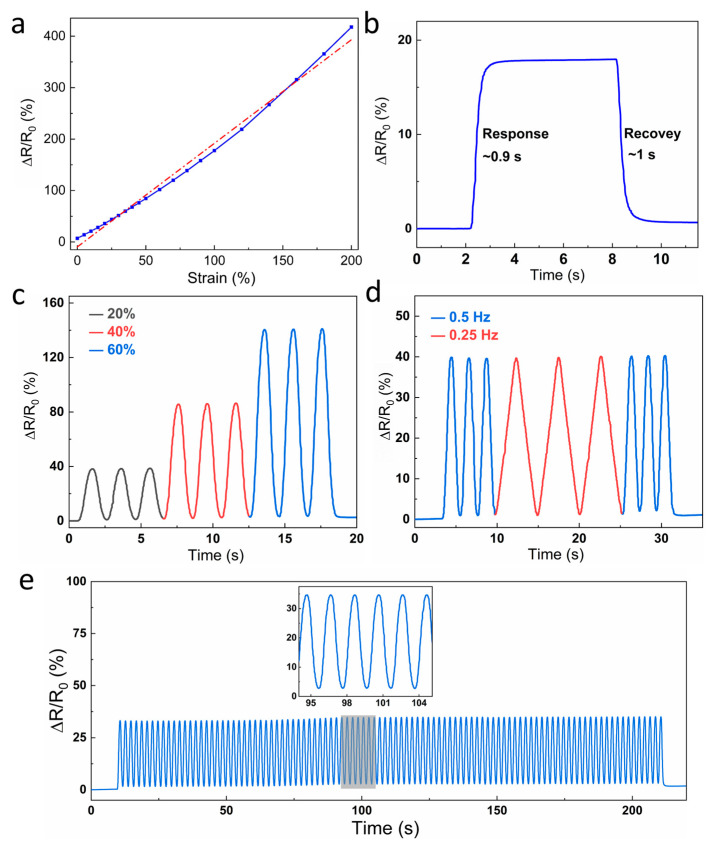
Sensing performance of the PPy/PA/PSBMA hydrogel as a strain sensor. (**a**) Electrical resistance and strain curves. The original data is represented by a blue line and the linear-fitted curve is represented by a red dashed line. (**b**) The response and recovery times of the hydrogel. (**c**) Real-time response curve measured at variable strains. (**d**) Real-time response curve measured at changeable frequencies. (**e**) Cycling durability of the hydrogel strain sensor.

Based on the outstanding performance of both electrical and mechanical properties, the as-prepared hydrogels were able to be used as strain sensors. Multiple tests were conducted using a PPy/PA/PSBMA hydrogel to characterize the performance of the hydrogel strain sensors (shown in Figure 6). The initial electrical resistance (R_0_) of the strain sensors was recorded at approximately 4.1 kΩ.

The gauge factor (GF) of the prepared strain sensor was measured and calculated to be ca. 2.01 in the range of 0~200% (shown in Figure 6a). The response and recovery times were measured to be 0.9 s and 1 s, respectively (shown in Figure 6b). In addition, the electrical resistance was almost the same before and after this process, exhibiting excellent stability as a strain sensor. Figure 6c illustrates the response curve recorded in real-time during three cycles at 20%, 40%, and 60% strains, while Figure 6d displays the real-time response curve at varying frequencies of 0.25 and 0.5 Hz. The cyclic stretching of the strain sensor resulted in repeatable and stable resistance variations, determined solely by the applied strains and independent of the stretching frequency. Afterwards, 100 repeated loading and unloading cycles were applied to the sensor with a frequency of 0.5 Hz. Negligible fluctuation of the response value could be observed, and the relative resistance variation was almost restored to zero every time after unloading the tensile stress (shown in Figure 6e), indicating the promising durability of the strain sensor. The stable and instant response can be attributed to the optimal combination of conductive PPy networks and elastic hydrogel networks.

**Figure 7:**
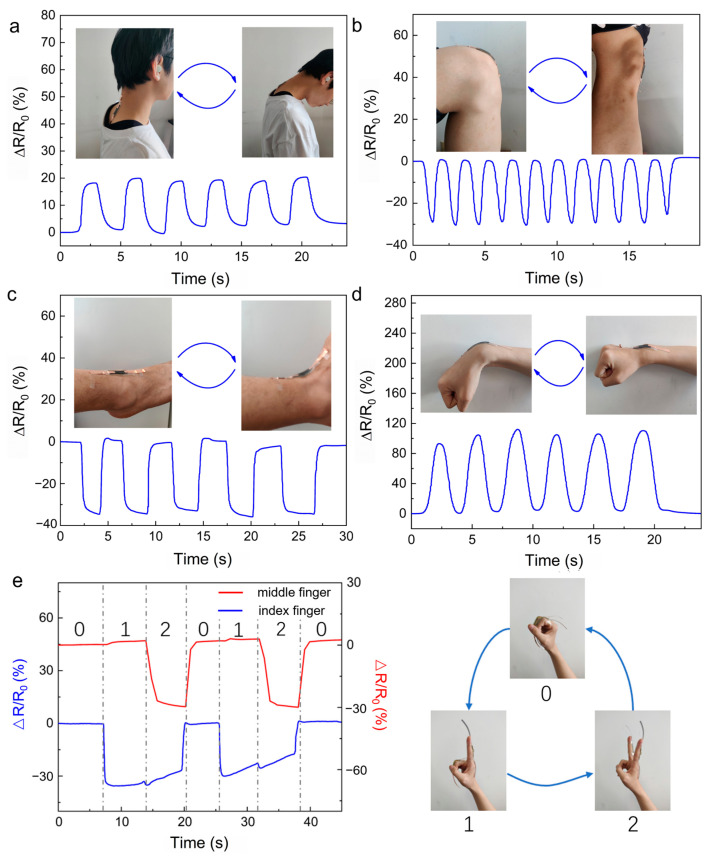
Application of the PPy/PA/PSBMA hydrogel for motion detection. The hydrogel could be used to monitor the movements of the (**a**) neck, (**b**) knees, (**c**) ankles, (**d**) wrists, and (**e**) the variations in gesture.

For further applications, an equipped strain sensor was fabricated for human motion detection. The prepared strain sensor could be used to monitor the movements of different parts during sport. The responses of the strain sensors are shown in Figure 7. The cyclic movements of the neck (Figure 7a), knees (Figure 7b), ankles (Figure 7c), and wrists (Figure 7d) caused changes in the expansion and contraction of the sensor, resulting in changes in electrical resistance. It is worth noting that repetitive movements produced stable wave-like responding signals. Moreover, the final response values nearly returned to zero after the movements, demonstrating the stability and efficiency of the as-prepared strain sensor. Furthermore, this strain sensor could be used to recognize the variations in gestures. If a finger were to be bent, the strain sensor would be stretched, resulting in an increase in resistance (shown in Figure 7e).

### 3.4. Extensibility of the One-Pot Preparation

**Figure 8:**
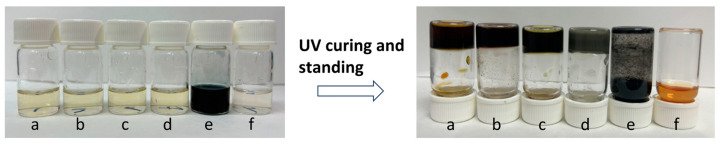
Trials of the hydrogel fabrication using different materials through the one-pot preparation method. (**a**) PPy/PA/PAAm. (**b**) PPy/PA/PAA. (**c**) PPy/PA/P (AAm-co-AA). (**d**) PPy/PA/PHEMA. (**e**) PPy/PA/PSBMA-APS. (**f**) PANI/PA/PSBMA.

For further research, we selected other commonly used hydrogel monomers for one-pot preparation of CPHs. The detailed materials are listed in Table 3. In the recipes, MBAA was employed as the cross-linking agent to facilitate the formation of elastic hydrogels. PA was added as a doping acid to enable the successful synthesis of PPy networks. H_2_O_2_ was used as a mild oxidant to initiate the polymerization of pyrrole. LAP or 2959 was used as photo initiator. It should be noted that, as a commonly used oxidant, APS is capable of initiating the polymerization reaction of both the hydrogel monomer and the CP monomer. The results are presented in Figure 8, responding to trials a–f.

As illustrated in Figure 8a–d, the CPHs based on polyacrylamide (PAAm), polyacrylic acid (PAA), P (AAm-co-AA), and poly-HEMA (PHEMA) were all successfully prepared, demonstrating the excellent extensibility of our one-pot procedure. The colors of these hydrogels eventually became dark, indicating the successful synthesis of PPy. Upon replacement of H_2_O_2_ into APS, a dark solution was obtained, demonstrating the absence of hydrogel formation (Figure 8e). Although APS could be used as a photo initiator for the polymerization of SBMA, it could also trigger the rapid polymerization of pyrrole. Therefore, the darkening of the solution impeded the photocuring of the hydrogels. In contrast to pyrrole, aniline was an inhibitor for the polymerization of SBMA (Figure 8f). Consequently, both the trials utilizing APS as an oxidant and aniline as a CP monomer were unsuccessful.

To further demonstrate that the other constructed CPHs also have a promising performance, similar experiments were conducted on the hydrogel based on P (AAm-co-AA) as an example. As illustrated in Figure S1 (in ESI), there are abundant hydrogen bonding interactions between the oxygen-containing moieties on PA molecules, carboxyl groups on AA, amide groups on AAm, and PPy chains. Likewise, electrical properties was investigated to further illustrate the formation of the PPy conductive networks. As shown in Figure S2 (in ESI), the electrical resistance of the hydrogels with PPy addition was apparently lower than that of the control samples without PPy. This reduction in electrical resistance was attributed to the additional intrinsic conductivity supplied by the PPy networks. Moreover, the PPy/PA/P (AAm-co-AA) hydrogel also has the potential to be employed as a strain sensor, displaying encouraging results (detailed exhibition is presented in Figure S3, in ESI).

## 4. Conclusions

In summary, we developed a facile and extensible one-pot processing procedure to prepare PPy-incorporated hydrogels for human motion sensing. Due to the use of a milder oxidant, H_2_O_2_, for PPy synthesis, the photocuring of the hydrogel matrix and the growing of PPy proceeded in an orderly manner, making it possible to prepare CPHs in one pot. Various CPHs based on different matrices could be prepared through this method.

The incorporation of PPy into hydrogels resulted in an enhanced conductivity compared to hydrogels without PPy. The as-prepared hydrogels possessed tunable mechanical properties adapted with human skin, and outstanding sensing stability. It was certificated that one-pot-prepared conductive hydrogels were well suited for human motion detection as strain sensors. We believe that our one-pot preparation strategy has great potential for the future development of advanced bioelectronic materials based on conductive hydrogels.

## Figures and Tables

**Table 1:** Conductivity of the PPy/PA/PSBMA hydrogels and the PA/PSBMA hydrogels.

Samples	PA (wt%)	Pyrrole (wt%)	Conductivity (mS/cm)
M_3_Py_5_	0.8	0.5	1.20
M_3_Py_10_	1.6	1	1.85
M_3_Py_15_	2.4	1.5	2.49
M_3_Py_20_	3.2	2	2.27
M_3_PA_5_	0.8	0	0.65
M_3_PA_10_	1.6	0	0.89
M_3_PA_15_	2.4	0	0.99
M_3_PA_20_	3.2	0	1.07

**Table 2:** Mechanical properties of the hydrogels with different cross-linking degrees.

Samples	MBAA (wt%)	Pyrrole (wt%)	Elongation at Break (%)	Young’s Modulus (kPa)
M_2_Py_15_	0.2	1	325	11.08
M_3_Py_15_	0.3	1	203	28.08
M_4_Py_15_	0.4	1	155	37.42
M_5_Py_15_	0.5	1	109	78.90

**Table 3:** Other recipes for hydrogels prepared using the facile one-pot method.

Recipes	Hydrogel Monomer	Photo Initiator	CP Monomer	Oxidizing Agent
a	AAm	2959	Pyrrole	H_2_O_2_
b	AA	LAP	Pyrrole	H_2_O_2_
c	AAm and AA	LAP	Pyrrole	H_2_O_2_
d	HEMA	2959	Pyrrole	H_2_O_2_
e	SBMA	APS	Pyrrole	APS
f	SBMA	2959	Aniline	H_2_O_2_

## Data Availability

The data presented in this study are available in the article.

## Supplementary Materials

The following supporting information can be downloaded at: https://www.mdpi.com/article/10.3390/s24175814/s1, Table S1: Materials and dosage of the PPy/PA/P (AAm-co-AAc) hydrogels and the PA/P (AAm-co-AAc) hydrogels; Figure S1: Various interactions among components of the PPy/PA/P (AAm-co-AA) hydrogel; Figure S2: The electrical resistance of the PPy/PA/P (AAm-co-AA) hydrogels and PA/P (AAm-co-AA) hydrogels; Figure S3: Sensing performance of the PPy/PA/P (AAm-co-AA) hydrogel as a strain sensor.
