# Supplementary material for: Facile One-Pot Preparation of Polypyrrole-Incorporated Conductive Hydrogels for Human Motion Sensing
Source: Sensors (Basel). 2024 Sep 7;24(17):5814. doi: 10.3390/s24175814 (PMC11397887; doi:10.3390/s24175814)
Supplement: Supplementary file 1 [file sensors-24-05814-s001.zip › sensors-3173200-supplementary.pdf]

Electronic Supplementary Information for

Facile One-Pot Preparation of Polypyrrole-Incorporated Conductive Hydrogels for Human Motion Sensing

Zunhui Zhao [1], Jiahao Liu [1], Jun Lv [2], Bo Liu [1], Na Li [1] and Hangyu Zhang [1,*]

[1] Liaoning Key Lab of Integrated Circuit and Biomedical Electronic System, School of Biomedical Engineering, Faculty of Medicine, Dalian University of Technology, Dalian 116024, China; zhao_zh@mail.dlut.edu.cn (Z.Z.); ljh2356486741@163.com (J.L.); lbo@dlut.edu.cn (B.L.); lina316@dlut.edu.cn (N.L.)

[2] School of Mechanics and Aerospace Engineering, Dalian University of Technology, Dalian 116024, China; lvjun@dlut.edu.cn

* Correspondence: hangyuz@dlut.edu.cn

This supplementary file includes:

Table S1

Figure S1 to S3

Table S1. Materials and dosage of the PPy/PA/P(AAm-co-AAc) hydrogels and the PA/ P(AAm-co-AAc) hydrogels.

Samples	AAm /mg	AA /mg	MBAA /mg	LAP /mg	50%PA/ mg	Py /mg	30%H_2O_2/ mg
$AAPy_{2.5}$	180	20	3	3	9.9	2.5	5.1
$AAPy_5$	180	20	3	3	19.8	5	10.2
$AAPy_{7.5}$	180	20	3	3	29.7	7.5	15.3
$AAPy_1$	180	20	3	3	39.6	10	20.4
$AAPA_{2.5}$	180	20	3	3	9.9	0	0
$AAPA_5$	180	20	3	3	19.8	0	0
$AAPA_{7.5}$	180	20	3	3	29.7	0	0
$AAPA_1$	180	20	3	3	39.6	0	0

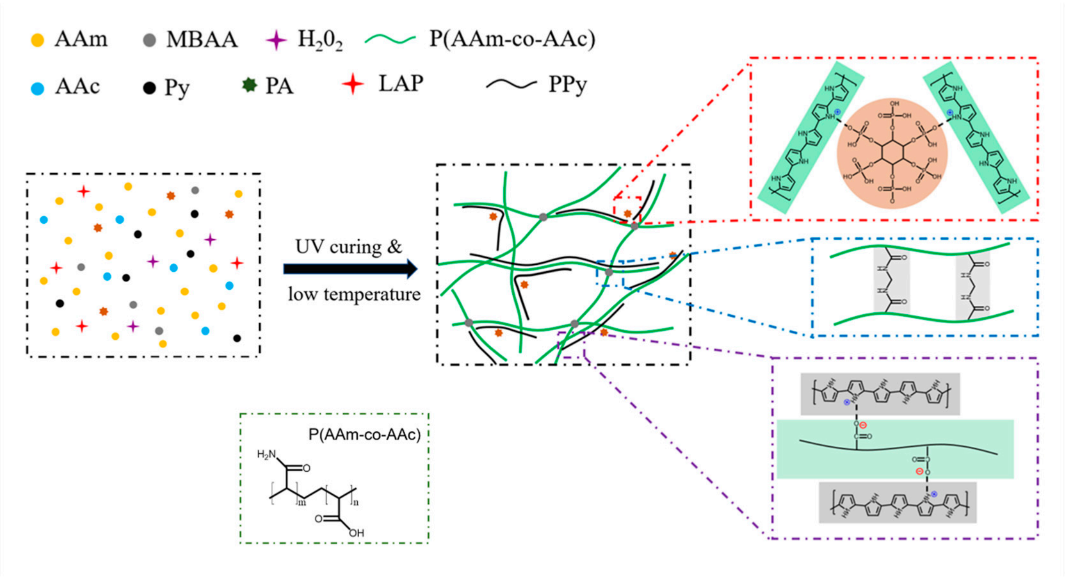

Figure S1. Various interactions among components of the PPy/PA/P(AAm-co-AA) hydrogel.

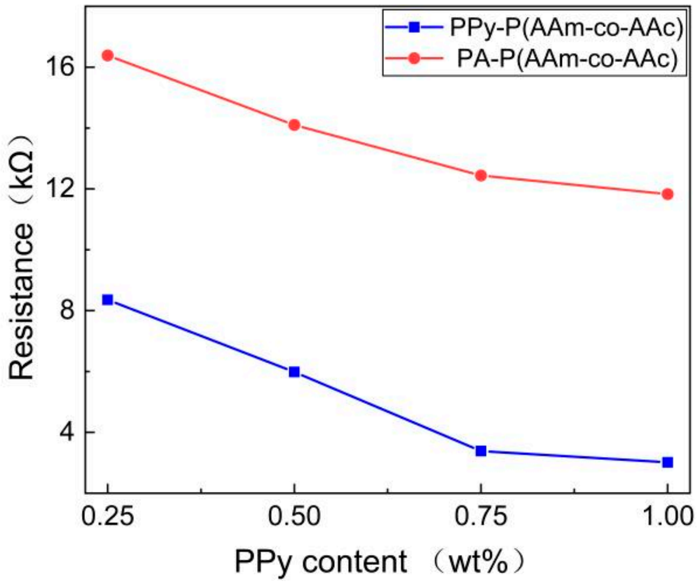

Figure S2. The electrical resistance of the PPy/PA/P(AAm-co-AA) hydrogels and PA/P(AAm-co-AA) hydrogels.

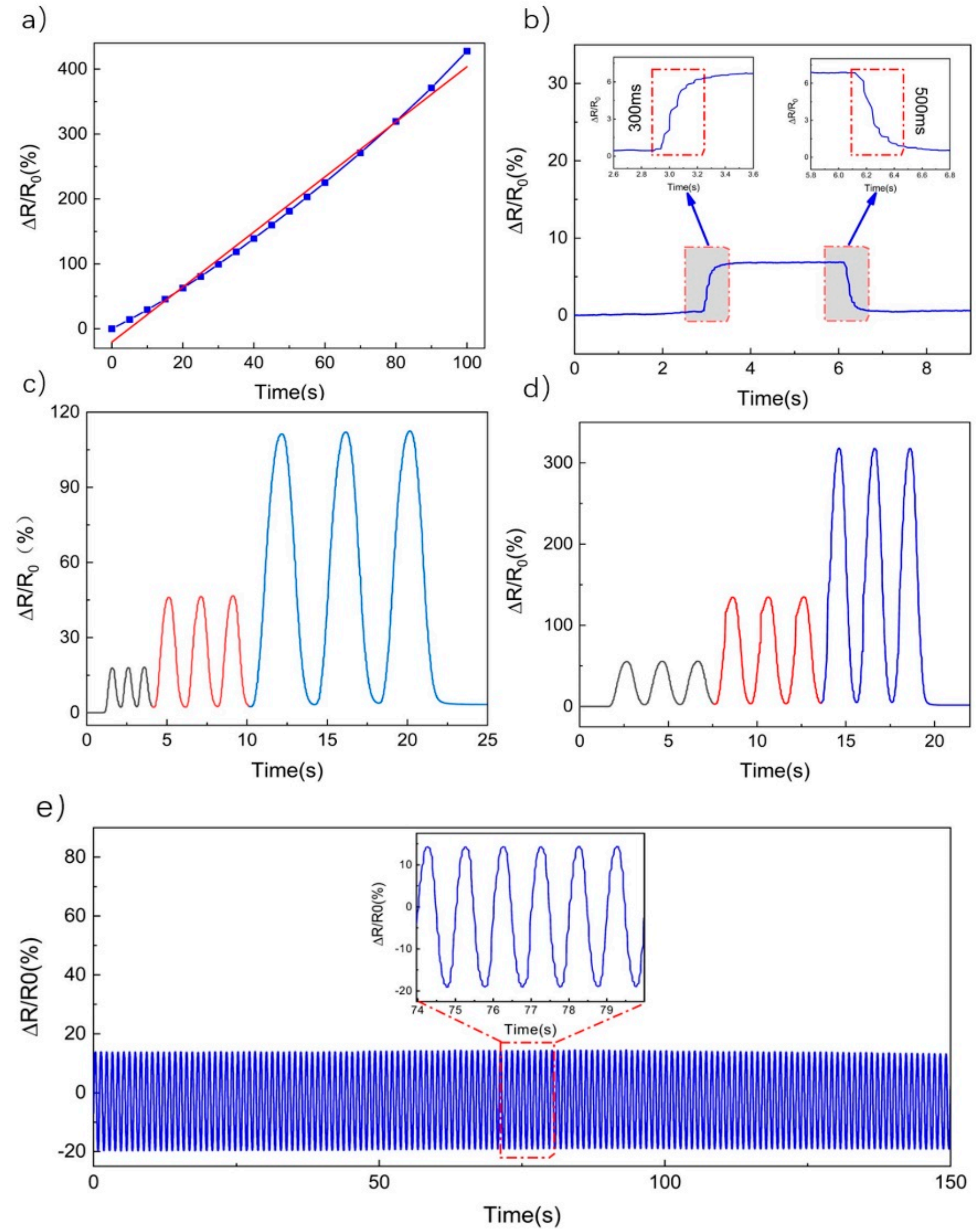

Figure S3. Sensing performance of the PPy/PA/P(AAm-co-AA) hydrogel as a strain sensor. (a) Electrical resistance and strain curves. (b) Response time of the hydrogel. (c)Real-time response curve measured at both changeable strains and changeable frequencies. (d) Real-time response curve measured at different strains. (e) Cycling durability of the hydrogel.
